# Positive and negative behavioural intentions towards immigrants: A question of ethnic categorisation or worldview conflict?

**DOI:** 10.1002/ijop.12748

**Published:** 2021-02-17

**Authors:** Willemijn Havermans, Maykel Verkuyten

**Affiliations:** ^1^ Ercomer Utrecht University Utrecht The Netherlands

**Keywords:** Prejudice, Immigrants, Ethnic boundaries, Worldview conflict, Authoritarianism

## Abstract

Anti‐immigrant attitudes are often explained in terms of ethnic boundaries in which a categorical distinction between the ethnic ingroup and immigrant outgroup is made. However, these attitudes might also result from contrasting cultural worldviews. We examined the importance of ethnic categorisation and perceived cultural worldview difference in explaining behavioural intentions towards immigrants. Using an experimental survey design with a national sample of ethnic Dutch respondents (*N =* 832), we studied two positive and two negative behavioural intentions towards either immigrants with a contrasting cultural worldview or co‐ethnics with such as worldview. Our findings indicate similar behavioural intentions towards both target groups. Furthermore, except for “the intention to learn” there were no differences in behavioural intentions towards both target groups for respondents with lower and higher authoritarian dispositions. Overall, this pattern of findings is theoretically most in line with a worldview conflict perspective rather than an ethnic boundary perspective.

International migration remains one of the central issues of our times with in 2019, around 272 million migrants worldwide. Immigrants are at the centre of political debates in many countries and anti‐immigrant attitudes are prevalent among majority members (Hainmueller & Hangartner, 2013; Hainmueller & Hopkins, 2014). Research in Western Europe and North America has found that especially concerns about incompatible cultural practices, norms and values are important for these negative attitudes, much more than economic concerns (Hainmueller & Hopkins, 2014; Sniderman & Hagendoorn, 2007). However, in this type of research respondents are typically presented with items that focuses on both the “object” that is at risk (one's culture) and the people (immigrants) that allegedly puts it at risk (e.g. “These days, I am afraid that our culture is threatened by immigrants”). This means that the measure might not only tap into feelings of cultural dissimilarity but also whether in general individuals dislike immigrants as an outgroup category. Hence, it is not fully clear what exactly drives people's responses (Spruyt & Elchardus, 2012).

On the one hand, people can respond to the category of immigrants with the related ethnic boundary drawing that defines immigrants as outsiders, “the ethnic other” (dislike of immigrants). Psychological research shows that there is a tendency to make ingroup (“us”) versus outgroup (“them”) distinctions whereby the outgroup is evaluated more negatively than the ingroup (Brown, 2010; Tajfel & Turner, 1979). Immigrants are people who have moved to another country in order to live there and their different ethnic background makes it likely that majority members make an ethnic ingroup versus outgroup distinction which contributes to a negative attitude towards immigrants.

On the other hand, people may respond negatively to cultural dissimilarities more generally rather than the category of immigrants per se (dislike of other cultural worldviews). People might be negative towards all others who are perceived as having a contrasting worldview from their own (Brandt & Crawford, 2020). This would mean that anti‐immigrant attitudes are not so much based on immigrants being an ethnic outgroup, but rather on the perception of cultural worldview conflict, regardless of the particular ethnic group.

In the current study conducted in the Netherlands we examine these two explanations by comparing behavioural intentions towards first generation immigrants (“newcomers”) and co‐ethnic majority members. We focus on behavioural intentions as the conative aspect of attitudes that is most closely to actual behaviour (Eagly & Chaiken, 1993). Using a national representative sample and an experimental survey design, ethnic^1^ Dutch participants were asked about their positive and negative behavioural intentions towards either immigrants with a contrasting cultural worldview or co‐ethnics with a contrasting worldview. Previous experimental research has varied the degree of cultural similarity to examine people's reactions towards culturally more and less similar immigrants (e.g. Sniderman & Hagendoorn, 2007; Spruyt & Elchardus, 2012). This allows to assess whether perceived cultural difference adds to the negative attitude towards the category of immigrants. In contrast, the current study varies the ethnic category for examining people's responses to those who have a contrasting cultural worldview. This makes it possible to assess whether being an immigrant adds to the negative attitude to culturally dissimilar people. Furthermore, we investigate the moderating role of individual differences in the key construct of authoritarian disposition (Feldman, 2003; Stenner, 2005) to further examine the ethnic boundary and the cultural worldview explanations for anti‐immigrant attitudes.

## Ethnic boundaries and worldview conflict

Extensive research has found that individuals are more likely to like, trust and favour people who are similar to themselves than people who are dissimilar (Byrne, 1971; McPherson et al., 2001). And research on social categorisation processes and social identity theory (Tajfel & Turner, 1979) has shown that making an ingroup versus outgroup distinction is sufficient for intergroup favouritism whereby people like, trust, help and sympathise more with ingroup than outgroup members. There is a large amount of empirical evidence that people tend to be parochial and spontaneously prefer their ingroup over relevant outgroups (e.g. Cicara & Van Bavel, 2014; Hewstone et al., 2002, for reviews). However perceived similarity can relate to different aspects and dimensions, including belonging to the same social category (social categorisation process) and having the same beliefs, norms and values (belief similarity). Ethnic boundaries create categorical distinctions based on perceived ethnic belonging but intergroup distinctions might also relate to worldview differences.

Individuals belong to various social categories that can intersect in different ways. Research on crossed categorisation proposes that the crossing of two categorisation dimensions leads to four possible combinations (see Crisp & Hewstone, 2007). Individuals can be crossed category members because they share category membership on one dimension but not on the other dimension (e.g. same ethnicity, but different worldview; different ethnicity, same worldview). Others can be double in‐group members because they share category membership on both dimensions (same ethnicity and same worldview), or double out‐group members by not sharing membership on any of the two categorisation dimensions (different ethnicity and different worldview). When both dimensions are equally relevant, crossed categorisation research has found a cumulative pattern of evaluations: double outgroup members are evaluated most negatively, double ingroup members most positively, and crossed category members are evaluated in between (see Crisp & Hewstone, 2007). In the current study we are interested in the question whether people make an ethnic category distinction given a perceived worldview difference: whether being an immigrant adds to the negative attitude to culturally dissimilar people. Therefore, we examine behavioural intentions towards co‐ethnics with a contrasting cultural worldview (crossed category) and immigrants with a contrasting cultural worldview (double outgroup). Following social categorisation reasoning this leads to the expectation that majority members show more negative and less positive behavioural intentions towards immigrants with a contrasting cultural worldview than towards co‐ethnics with such a worldview.

However, a contrasting thesis can be derived from the worldview conflict perspective as developed in social psychology (Brandt & Crawford, 2020). Similar to integrated threat theory (Stephan & Stephan, 2000) and the many studies on the importance of symbolic threat for prejudicial attitudes (see Brown, 2010), this perspective proposes that people have a desire for a consistent cultural worldview which they want to maintain and defend against worldview‐threatening others. As a result people tend to be negative towards others whose values and norms conflict with, or threaten, one's own norms and values (Brandt & Crawford, 2020). Substantial differences in norms and values are a particularly pronounced and potent form of dissimilarity that can override ethnic categorical distinctions. The perception that others hold cultural belief systems incompatible with one's own can be more important for social discrimination than mere ethnic belonging (Insko et al., 1983; Rokeach, 1960). This means that people can have negative attitudes towards others with opposing norms and values, independent of the ethnic category these others belong to.

This suggestion is in agreement with findings of crossed categorisation research in which one dimension is more relevant or important than the other (see Miller et al., 2006). In such a situation the group distinction on the more important category dimensions (i.e. worldview) exerts more influence on people's attitudes and behaviours than the distinction on the less important dimension (ethnicity). Thus when the worldview difference is more important than the ethnic boundary, the evaluation of people who only differ in their worldview (ethnic Dutch with different norms and values) is lowered to the level of people who differ on both dimensions (immigrants with a different worldview). This leads to the contrasting thesis that majority members do not show more negative and less positive behavioural intentions towards immigrants with a contrasting cultural worldview than towards co‐ethnics with such a worldview.

## The role of authoritarian disposition

An additional way to examine the two contrasting expectations is to consider the role of individual differences in authoritarian disposition which is a core psychological construct for the explanation of prejudice towards minority groups and immigrants (e.g. Craig & Richeson, 2014). Following the original formulation of authoritarianism and its subsequent reconceptualisation (Altemeyer, 1998), recent conceptualisations are based on the notion of a general underlying tension between the goals of personal autonomy and social conformity (Feldman, 2003; Stenner, 2005). Specifically, authoritarians are considered to emphasise and value conformity and obedience over self‐direction and independence (Feldman, 2003; Stenner, 2005). Authoritarians' tend to feel aversion towards minority others that are dissimilar and unfamiliar to them (Stenner, 2005; Van Assche et al., 2014). Research has shown that authoritarianism strongly correlates with prejudice towards outgroups that are considered normatively threatening, such as sexual minorities (Cohrs & Asbrock, 2009), drug dealers (Cohrs & Ibler, 2009), and immigrant groups (e.g. CohrsCohrs & Ibler, 2009; Kauff et al., 2015). Authoritarians favour assimilation of immigrants and they are especially upset by immigrants who are perceived to be different (Thompson et al., 2008). Additionally, authoritarians tend to identify strongly with their national ingroup and belief in the superiority of their nation, which makes it likely that they perceive immigrants as ethnic outsiders (e.g. Blank, 2003; Osborne et al., 2017; Stenner, 2005). The implication could be that higher, compared to lower, authoritarians will respond more negatively towards immigrants with a contrasting cultural worldview than towards co‐ethnics with such a worldview (moderation effect of authoritarianism).

The alternative thesis derived from the worldview conflict perspective is that people with a low or high authoritarian disposition are equally negative towards both co‐ethnics and immigrants with a contrasting worldview. People, in general, might be negative towards those with opposing norms and values. For example, research has found that individuals who are either high or low on openness to experience, who are high or low on agreeableness, or high or low on conscientiousness are all prejudiced towards others whose worldviews conflict with their own, and that people with high or low levels of religious fundamentalism are negative towards those with dissimilar religious beliefs (Brandt & Van Tongeren, 2017; Kossowska & Sekerdej, 2015). Furthermore, whereas individuals with lower cognitive ability are prejudiced towards liberal and unconventional groups, those with higher cognitive ability tend to be prejudiced towards conservative and conventional groups (see Brandt & Crawford, 2020). Thus, belief dissimilarity can be the main driver of outgroup negativity, independently of important individual differences. This might mean that both higher and lower authoritarians do not differentiate in their positive and negative behavioural intentions towards immigrants with a conflicting cultural worldview and co‐ethnics with such a worldview (no moderation effect of authoritarianism).

## Method

### Sample

This research makes use of data from the Social Integration Survey^2^ that has been conducted in the Netherlands in February, 2019. Ethnic Dutch participants were selected^3^ from the national representative panel maintained by research organisation *Ipsos*, and a random sample of 1640 panel members was approached. The response rate was 52%^4^ (*N* = 832) which is similar to other national surveys in the Netherlands (Stoop et al., 2010). Participants were all Dutch citizens and had a mean age of 54.83 (*SD =* 16.24, range = 18–88), and 50.5% (*N =* 420) was female. Participants were informed that participation was voluntary and anonymous and received the regular compensation (e.g. bonus points) as part of their continuing involvement in the panel.

### Design and measures

Participants completed a survey online and were randomly assigned to one of the two experimental conditions that were relevant for this study. They were asked questions related to either the target categories of “newcomers (first generation immigrants)” (*N* = 415) or “ethnic Dutch” (*N* = 417) with contrasting cultural worldviews: “The next questions are about your reactions towards newcomers in the Netherlands [autochthonous Dutch^5^] who compared to you have very different beliefs, norms and values and thus live very differently. How likely is it that you react to them in the following ways?”

Subsequently participants were presented with a list of positive and negative behavioural intentions. We focused on behavioural intentions because these tend to be closer to actual behaviour than stereotypes, evaluations and feelings (Eagly & Chaiken, 1993). Furthermore, we distinguished between positive and negative behaviours because the intention to engage in positive behaviour does not necessarily imply a willingness to engage in negative behaviour, and vice versa (Lalljee et al., 2009). We used 12 items (7‐point scales, 1 = “I would never do that,” 7 = “I would certainly do that”) based on Lalljee et al. (2009). The items for negative behavioural intentions tap into two sub‐constructs with three items each: aggression (“confront them,” “oppose them” and “argue with them”) and avoidance (“keep them at a distance,” “avoid them” and “go out of their way”). The items for positive behavioural intentions also focus on two aspects with three items each: learning (“learn from them,” “spend time with them” and “find out more about them”) and tolerating (“endure their way of life,” “accept their way of life” and “tolerate their way of life”).

*Authoritarian disposition* was measured at the beginning in the same online survey and in a separate section before the experimental manipulation. We used an extended version of the “child‐rearing preference” measure. This measure was designed to tap into the underlying disposition by assessing a relative priority and therefore creates a trade‐off between stimulating social conformity and obedience versus self‐direction and autonomy in socialising children (Feldman, 2003; Stenner, 2005). The items do not reference any social groups, or political events and actors which means that the scale is not tautological with the outgroup attitudes and behaviours that one wants to explain (Stenner, 2005). Respondents were presented with four pairs of qualities children could be taught (e.g. obeying parents versus making one's own choices) and for each of the pairs they were asked which one they consider to be more important. Subsequently, they were asked to indicate how much more important they found this quality using a 3‐point scale (slightly more important, more important, or much more important). The answers to the questions for a given pair of qualities were recoded to a six‐point scale so that a higher score indicates stronger authoritarian disposition (ρ = .72).

*Education* was measured on a 7‐point ordinal scale, ranging from *“*no education/only lower education/integration course/Dutch language course” (1) to “Doctoral or master's degree or postgraduate education” (7). Similar to other research in the Netherlands (e.g. De Graaf et al., 2000; Van Tubergen & Van de Werfhorst, 2007), education was treated as a continuous variable in the analysis. Participants were asked to indicate their political preference on a 7‐point self‐placement scale (Jost, 2006) ranging from “*strongly left”* (1) to “*strongly right”* (7), and *national identification* was measured with a valid single item (Postmes et al., 2013): “How strongly do you feel Dutch?”

### Analysis

The analyses were conducted in Mplus Version 7.3 (Muthén & Muthén, 2010). With confirmatory factor analysis it was first examined whether the items of the different measures loaded on separate latent constructs. The measurement models were fitted using structural equation modelling with the estimator MLR (maximum likelihood estimation with robust standard errors) to accommodate non‐normality of the measures. Subsequently we tested for measurement invariance of the behavioural intention items across the two experimental conditions. Second, we tested our hypothesis using Structural Equation Modelling in Mplus. To examine whether the measurement and structural models fitted the data properly, common fit statistics, such as Tucker–Lewis Index (TLI), comparative fit index (CFI), the root mean square error of the approximation (RMSEA), and Akaike Information Criterion (AIC) were used (Satorra & Bentler, 2010).

The two experimental groups did not differ significantly (*p*_*s*_ > .095) for gender, age, education, political orientation, national identification and authoritarianism. This indicates that the randomisation was successful and that the ethnic Dutch participants in the two experimental conditions did not differ in these demographics and measured constructs. However, because these factors have been found to be associated with attitudes towards immigrants (see Ceobanu & Escandell, 2010), we controlled for these factors in an additional analysis to assess whether this changed the experimental findings.

## RESULTS

### Measurement model and descriptive findings

A Confirmatory Factor Analysis was conducted to examine the fit of a model with the five measured constructs: aggression, avoidance, learning, tolerating, and authoritarianism. This model had a good fit to the data, χ^2^(94) = 284.98, *p* < .001; CFI = 0.97; TLI = 0.96; RMSEA = 0.049; AIC = 38,824.39, with standardised factor loadings above .72 (see Kline, 2016).

Additionally, in order to assess whether the behavioural intention items had the same meaning in relation to the two targets groups (immigrants or ethnic Dutch), measurement invariance across the two experimental conditions was assessed (see Appendix and Table A1). We found full invariance which indicates that the latent constructs had similar meanings in relation to the two target groups. Furthermore, the variances and covariances of the latent variables were equal across the two target groups because we could not reject the assumption of equal variances, *Wald*(4) = 8.023, *p =* .091. The associations between the different latent constructs can be found in Tables 1 and 2 the descriptive findings of the latent variables and for the two experimental conditions are shown.

**Table 1:** Correlations between the latent variables

Latent variables	Aggression	Avoidance	Learning	Tolerating
Aggression				
Avoidance	.08			
Learning	.22***	−.79***		
Tolerating	−.06	−.60***	.88***	
Authoritarianism	.01	.18**	−.23***	−.32***

*Note*. Correlations of latent variables were obtained from Mplus using the effect‐coding method of model identification for latent variables (Little et al., 2006).

** *p* < .01.

*** *p* < .001.

**Table 2:** Means and standard deviations of the latent variables for the two experimental target groups of Ethnic Dutch and Immigrants

	Ethnic Dutch (n = 415)		Immigrants (n = 417)	
Target groups	M	SD	M	SD
Negative behavioural intentions				
Aggression	3.38	1.14	3.34	1.13
Avoidance	3.06	1.37	2.99	1.43
Positive behavioural intentions				
Learning	3.92	1.11	4.07	1.22
Tolerating	4.33	1.09	4.38	1.27
Authoritarianism	4.01	.88	3.98	.87

*Note*. Descriptives of latent variables were obtained from Mplus using the effect‐coding method of model identification for latent variables (Little et al., 2006).

### Behavioural intentions

We performed Structural Equation Modelling (SEM) using Maximum Likelihood Estimation with robust standard errors (MLR) in Mplus to examine differences in behavioural intentions towards immigrants and the ethnic Dutch. We first fitted a model in which the four behavioural intentions were regressed on the experimental condition and authoritarianism. The main effects are presented in Model 1 (M1) in Table 3. None of the behavioural intentions differed significantly between the two conditions. Further, people higher on authoritarianism were in general less willing to learn, less tolerant and more avoiding, but not more aggressive towards others with a contrasting worldview.

**Table 3:** Unstandardised coefficients of a model with main effects (M1), a model including the interaction effect (M2), and a model including control variables (M3) (*N* = 832)

	Negative intentions						Positive intentions					
	Aggression			Avoidance			Learning			Tolerating		
	M1	M2	M3	M1	M2	M3	M1	M2	M3	M1	M2	M3
Main effects												
Exp. condition	−.030 (.083)	−.030 (.083)	−.029 (.080)	−.055 (.088)	−.055 (.088)	−.041 (.086)	.114 (.078)	.114 (.078)	.090 (.075)	.025 (.080)	.025 (.080)	−.013 (.076)
Authoritarianism	.008 (.055)	−.089 (.074)	−.142 (.079)	.190 (.057)***	.103 (.079)	.024 (.083)	−.237 (.051)***	−.109 (.065)	−.014 (.071)	−.365	−.281 (.069)***	−.117 (.068)
Interaction effect												
Condition × Author.		189 (.106)	.179 (.105)		.183 (.111)	.154 (.111)		−.253 (.096)**	−.222 (.094)*	(.054)***	−.171 (.099)	−.154 (.096)
Control variables												
Gender (male = 0)			−.505 (.082)***			−.173 (.088)			.014 (.075)			−.005 (.078)
Age			.011 (.003)***			−.006 (.003)*			.010 (.002)***			.006 (.003)*
Education			.001 (.027)			−.024 (.029)			.077 (.026)**			.145 (.025)***
Political orient.^a^			.038 (.032)			.175 (.038)***			−.202 (.033)***			−.200 (.032)***
National ident.^a^			.008 (.026)			.013 (.026)			.036 (.027)			.048 (.024)*

*Note*. Structural equation modelling using full information maximum likelihood estimation in Mplus version 8. Unstandardised coefficients reported with standard error in brackets. Two tailed significance tests.

* *p* < .05.

** *p* < .01.

*** *p* < .001.

a Political orientation (“left to right”; *N =* 737) and national identification (*N* = 830) were endogenised which led to a lower *N* on these two variables.

In order to examine the moderating role of authoritarianism, we added an interaction term between authoritarianism and experimental condition in a second model (see M2 in Table 3). There was only a significant interaction effect for learning. Simple slope analyses showed that the association between authoritarianism and learning intention was more strongly negative in relation to immigrants (*B* = −.32, *se* = .058, *t* = 5.551, *p* < .001) than co‐ethnics (*B* = −.11, *se* = .057, *t* = 1.902, *p* = .058). Participants with lower scores on authoritarian disposition wanted to learn more from immigrants than from co‐ethnics, whereas higher authoritarians did not differentiate in their learning intentions between immigrants and ethnic Dutch with a contrasting cultural worldview.

In a final step, we added the control variables to the model (M3), which did not change in the findings.

## DISCUSSION

Survey and experimental research has found that perceived cultural incompatibilities and the related cultural threats are important for anti‐immigrant attitudes (e.g. Hainmueller & Hopkins, 2014; Sniderman & Hagendoorn, 2007). However, in survey research participants are typically presented with two types of information: on the category of immigrants and on cultural differences. This means that the measure might not only tap into feelings of cultural threat but also whether people reject immigrants because they are an ethnic outgroup. Experimental research has also examined whether a larger perceived cultural difference makes people more negative towards immigrants (Sniderman & Hagendoorn, 2007; Spruyt & Elchardus, 2012), but this does not allow to draw conclusion about the importance of social categorisation processes and the related ethnic boundaries separating majority members and immigrants.

In the current study we examined the contrasting predictions derived from processes of social categorisation (Tajfel & Turner, 1979) and perceived worldview conflict (Brandt & Crawford, 2020). Ethnic Dutch majority members were asked to indicate their intentions towards either newcomers with a contrasting cultural worldview or co‐ethnics with such a worldview, and we did not find any significant differences in positive and negative behavioural intentions towards the two target groups. Thus, participants were equally positive and negative towards culturally dissimilar immigrants as they were to culturally dissimilar co‐ethnics. This pattern of findings does not support an ethnic ingroup versus outgroup categorisation interpretation but is in line with the worldview conflict proposition (Brandt & Crawford, 2020).

Furthermore, we found only very limited support for the expectation that people with a stronger authoritarian disposition differentiate more in their positive and negative intentions towards culturally dissimilar co‐ethnics and towards immigrants. For tolerance, avoidance and aggression intentions, we did not find a significant interaction effect. This is in agreement with worldview conflict research that does not find differences in attitudes towards others with a contrasting worldview between individuals who are high or low on openness to experience, high or low on agreeableness, high or low on conscientiousness, high or low on cognitive ability, and have high or low levels of religious fundamentalism (see Brandt & Crawford, 2020). Rather people, in general, tend to respond towards others with opposing worldviews at all levels of these individual difference variables, and thus also at different levels of authoritarian disposition. This is in agreement with a line of research on dissimilarity‐prejudice effects (Byrne, 1971; Rokeach, 1960) and suggests that also people with a low authoritarian disposition are not immune to the effects of perceived worldview conflict and are inclined to engage in forms of worldview defensive behaviour. Yet, we did find a small significant interaction effect for learning intentions whereby individuals with a lower authoritarian disposition demonstrated a higher intention to learn from culturally dissimilar immigrants than from co‐ethnics. One possible reason for this finding is that learning requires a more active engagement with culturally others, which lower authoritarians might be more motivated to do in relation to immigrants than co‐ethnic majority members.

It is important to note that we measured authoritarianism as an underlying disposition using the child‐rearing preference format (Feldman, 2003; Stenner, 2005). The advantage of this measure, compared to for example the Right‐Wing Authoritarianism scale (Altemeyer, 1998), is that the items do not refer to specific social groups or a particular social and political context. The usefulness of the measure is demonstrated in the significant direct associations that we found with the positive and negative behavioural intentions. Yet, future research could extend the current findings by using other measures of authoritarianism and also by focusing on other facets of authoritarianism such as moral absolutism, suppressed aggression, and submission to established authority.

We focused on contrasting cultural worldviews because this is how immigrants are often portrayed in the media and in political discourses. However, ethnic boundaries might become more relevant in relation to immigrants that are considered to have very similar cultural norms and values as co‐ethnics. Hence, the current research can be extended by using an experimental design in which not only the ethnic category is manipulated but also the degree of cultural worldview difference. Specifically, people can also be asked about their behavioural intentions towards immigrants with a similar cultural worldview. This would allow to further determine the relative importance of both ethnic categorisation and perceived worldview difference for people's attitudes (Hainmueller & Hopkins, 2014). Furthermore, future research could examine the robustness of the current findings by focusing on attitudes towards specific immigrant groups and immigrant generations and could also examine what sort of subgroups people have in mind when thinking about immigrants (Braun et al., 2019; Spruyt & Elchardus, 2012).

Future research could also examine whether the current findings generalise to other national contexts. Cross‐national research shows that the national context can play a role in shaping inter‐ethnic relations and attitudes towards immigrants (e.g. Guimond et al., 2014; Phalet & Baysu, 2020). Not all countries exhibit negative attitudes towards immigrants to the same degree (Heath & Richards, 2020). A country's history, political constellation, immigration and integration policies, and national self‐understanding might all matter for people's attitudes. However, research has also found, for example, that in many countries attitudes towards immigration are shaped by concerns about the cultural impact of immigration (Hainmueller & Hopkins, 2014) and feelings of threat (Valentino et al., 2019), and that more conservative people are more negative towards immigrants (e.g. Semyonov et al., 2008). Country differences in public attitudes do not necessarily mean that the relevance of various psychological processes also differ, although the strength of the processes might vary. For example, it is likely that ethnic boundaries are more important for people's attitudes in societies in which immigrants are discussed in relation to ethnic versus civic nationhood and in societies in which the relative size of the immigrant population is larger.

In conclusion, among a national sample we found that people demonstrate equally positive and negative behavioural intentions towards newcomers with a contrasting cultural worldview as towards co‐ethnics with such a worldview. This indicates that the ethnic ingroup versus outgroup distinction does not add to people's reactions towards immigrants when a contrasting worldview is involved. Rather, the perceived cultural dissimilarity seems to overpower the ethnic boundary, making people, in general, wanting to engage in behaviours that protect the continuity and validity of their own cultural belief system.

## Supporting information

**Appendix S1:** Supporting Information.

## Endnotes

^1^
Here we use the term “ethnic” in the sense of the belief in common descent and ancestry which goes back to the sociologists Max Weber (Cornell & Hartmann, 1988). In the Dutch context the term has the advantage that the “native” Dutch are considered an ethnic group similar to other ethnic (minority) groups, rather than the default. In the Dutch context one uses, for example, terms such Turkish‐Dutch and Moroccan‐Dutch but not Dutch–Dutch because the label for the majority group is similar to the one for the national group.

^2^
Ercomer, Utrecht University. The data are securely stored at the special data storage facility of Utrecht University. The measures and data will be made available through the open science framework, https://osf.io

^3^
The selection is based on the definition used in demographic research conducted by the Central Statistical Office of the Netherlands. It implies that both parents have been born in the Netherlands which is not the case for the great majority of immigrant‐origin adults. Additionally, at the start of the survey participants were asked whether they considered themselves of Dutch ethnic origin and all participants did so.

^4^
Ipsos, research report, March 7th, 2019.

^5^
In the Netherlands, to identify the ethnically Dutch the term “autochthonous” is typically used in everyday language and was therefore used in the question.
