# Supplementary material for: Positive and negative behavioural intentions towards immigrants: A question of ethnic categorisation or worldview conflict?
Source: Int J Psychol. 2021 Feb 17;56(5):633–41. doi: 10.1002/ijop.12748 (PMC8451776; doi:10.1002/ijop.12748)
Supplement: Supplementary file 1 — Appendix S1: Supporting Information. [file IJOP-56-633-s001.docx]

**Appendix**

Measurement invariance between the two experimental groups (immigrants vs. co-ethnics)

First, we tested for configural invariance and obtained an adequate model fit, meaning that the measurement of the four behavioral intentions is valid for both experimental conditions (see Table A1). We continued to test for metric invariance, by constraining the factor loadings to be equal across both conditions. The metric model was not significantly worse than the configural model, which indicates that individuals in both experimental conditions attribute the same meaning to the latent constructs (Van de Schoot, Lugtig & Hox, 2012). We continued by constraining the intercepts to be equal across the two groups to see whether we could meet scalar invariance. Again, this model turned out to not significantly worse than the metric model, indicating that the scores on the latent variables can be compared across the two conditions. Lastly, we fitted a model in which the residual variances were fixed to be equal across the conditions. The full invariance model was not significantly worse than the scalar model, meaning that the latent constructs are identically measured across the two experimental conditions.

Table A1

*Measurement invariance models*

| Invariance Models | MLR χ2 | df | RMSEA | CFI | TLI | SB scaled Δχ^2^ | Δdf | *p* |
| --- | --- | --- | --- | --- | --- | --- | --- | --- |
| M1 Configural | 249.279 | 96 | .062 | .969 | .957 |  |  |  |
| M2 Metric | 254.296 | 104 | .059 | .969 | .961 | 4.316 | 8 | .828 |
| M3 Scalar | 266.302 | 112 | .058 | .968 | .963 | 10.096 | 8 | .258 |
| M4 Full | 267.895 | 124 | .053 | .970 | .969 | 13.447 | 12 | .337 |

*Note:* MLR χ2 = Santorra-Bentler Chi Square test, df = Degrees of freedom, RMSEA = Root Mean Square Error of Approximation, CFI = Comparative Fit Index, TLI = Tucker-Lewis coefficient, SB scaled Δχ^2^ = Santorra-Bentler Chi Square difference test.
